# High‐resolution quantitative proteome analysis reveals substantial differences between phagosomes of RAW 264.7 and bone marrow derived macrophages

**DOI:** 10.1002/pmic.201400431

**Published:** 2015-02-05

**Authors:** Manman Guo, Anetta Härtlova, Brian D. Dill, Alan R. Prescott, Marek Gierliński, Matthias Trost

**Affiliations:** ^1^ MRC Protein Phosphorylation and Ubiquitylation Unit, College of Life Science, University of DundeeScotlandUK; ^2^ Division of Cell Signalling and Immunology, College of Life Science, University of DundeeScotlandUK; ^3^ Division of Computational BiologyCollege of Life SciencesUniversity of DundeeScotlandUK

**Keywords:** Cell biology, Macrophage, Phagosome, Proteomics RAW 264.7

## Abstract

Macrophages are important immune cells operating at the forefront of innate immunity by taking up foreign particles and microbes through phagocytosis. The RAW 264.7 cell line is commonly used for experiments in the macrophage and phagocytosis field. However, little is known how its functions compare to primary macrophages. Here, we have performed an in‐depth proteomics characterization of phagosomes from RAW 264.7 and bone marrow derived macrophages by quantifying more than 2500 phagosomal proteins. Our data indicate that there are significant differences for a large number of proteins including important receptors such as mannose receptor 1 and Siglec‐1. Moreover, bone marrow derived macrophages phagosomes mature considerably faster by fusion with endosomes and the lysosome which we validated using fluorogenic phagocytic assays. We provide a valuable resource for researcher in the field and recommend careful use of the RAW 264.7 cell line when studying phagosome functions. All MS data have been deposited in the ProteomeXchange with identifier PXD001293 (http://proteomecentral.proteomexchange.org/dataset/PXD001293).

**Abbreviations**
BMDMs: bone marrow‐derived macrophages
MRC1: mannose receptor 1

Macrophages are immune cells that exist in many different tissues and perform a wide range of biological functions 1, 2. They are extremely plastic in their protein expression pattern and can become activated by a range of cytokines and pathogen‐associated molecules such as lipopolysaccharide. One of the main functions of macrophages is phagocytosis, which is the active uptake of large particles (> 0.5 μm) by cells 3. Phagocytosis is an important cellular mechanism for almost all eukaryotes, highly conserved from amoeba to human 4, in mammals, it is a key part of the innate immune response against invading microorganisms. Moreover, macrophages phagocytose apoptotic cells and cell debris to recycle cellular building blocks during homeostasis and development 5, 6. Phagocytosis is induced through the binding of particles to cell surface receptors such as the mannose receptor, Fc‐receptors, or scavenger receptors. After internalization, newly formed phagosomes engage in a maturation process that involves fusion with endosomes, lysosomes, and other organelles 7, 8 leading to the formation of phagolysosomes in which the foreign matter is degraded. Peptide antigens from the particle are also presented via MHC class I and II molecules, bridging innate and adaptive immunity.

The macrophage‐like cell line RAW 264.7 9 was derived from Balb/c mice injected with Abelson murine leukemia virus and has been extensively used in the phagocytosis and macrophage field in the past with almost 4000 articles listed in Pubmed in 2014. In recent years, the cell line has also been used extensively in proteomics experiments including a large‐scale proteome 10, phagosome proteomics 4, 8, 11, responses to cytokines 12, 13, and identification of DNA receptors 14.

In this paper, we have compared the phagosomal functions and proteomes of RAW 264.7 and bone marrow derived macrophages (BMDMs) 15, 16 in order to determine if the cell line was a good substitute for primary macrophages when studying phagosome and macrophage biology.

First, we performed a set of experiments to characterize differences in phagocytic uptake of beads and phagosome maturation in RAW 264.7 and BMDMs. Phagocytic uptake assays using Alexafluor488‐labeled 3 μm silica beads showed a significantly reduced phagocytosis rate in RAW 264.7 compared to BMDM, particularly at early time points (Fig. 1A). Moreover, we performed fluorogenic assays to measure pH, proteolysis, and oxidative capacity of phagosomes in real time 17, 18, 19 and identified a significant faster phagosome acidification in BMDMs (Fig. 1B), which is possibly due to the faster phagocytosis compared to RAW 264.7 cells. However, BMDMs also display a much stronger proteolytic activity, suggesting a quicker fusion with lysosomes, a reduced inactivation of lysosomal proteases, and/or a higher concentration of proteases in the lysosome (Fig. 1C). Interestingly, RAW 264.7 cells produce a similar or even slightly higher oxidative burst in phagosomes compared to BMDMs (Fig. 1D), suggesting that this function is well conserved in the cell line.

**Figure 1:**
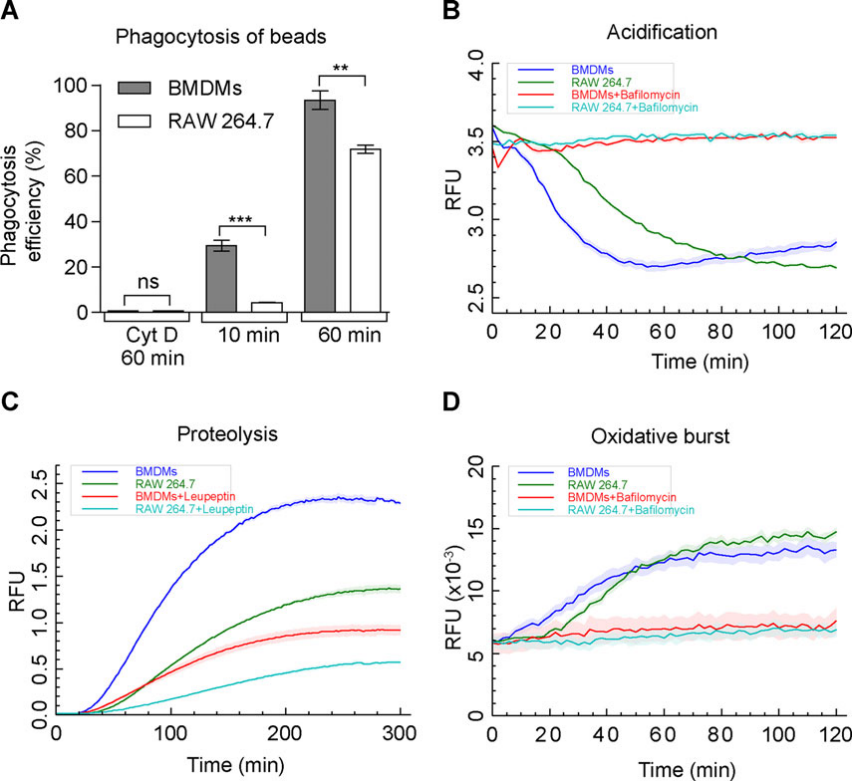
BMDMs display an increased rate of phagocytosis, phagosomal acidification, and proteolysis compared to RAW 264.7 macrophages. (A) BMDMs show a strongly increased efficiency of phagocytosis of AF‐488‐coated 3 μm beads compared to RAW 264.7 cells. Cells treated with the actin polymerization inhibitor cytochalasin D were used as nonphagocytic negative controls. Error bars represent standard deviation, pair‐wise *t*‐test comparison with ***p < 0.0001 and **p < 0.001. (B–D) Real‐time kinetics of fluorescent phagosome assays. BMDM phagosomes acidify significantly faster (B), display a much faster and stronger phagosomal proteolysis (C) but a similar oxidative burst (D) compared to RAW 264.7 cells. Shaded areas around lines are the standard error of mean (SEM) of six replicates.

In order to get insights into the molecular differences of the RAW 264.7 and BMDM phagosome proteomes, we performed a label‐free proteome analysis of phagosomes from both cell types. Phagocytosis was induced for 30 min using carboxylated 1 μm polystyrene beads (Estapor/EMD Millipore) and phagosomes were isolated using sucrose ultracentrifugation in which the phagosomes float above cell debris and other organelles 11, 20. Protein extracts from three biological replicates were resuspended in 1% sodium 3‐[(2‐methyl‐2‐undecyl‐1,3‐dioxolan‐4‐yl)methoxy]‐1‐propanesulfonate (commercially available as RapiGest, Waters) in 50 mM Tris‐HCl pH 8.0 with 5 mM tris(2‐carboxyethyl)phosphine (TCEP, Pierce), heated at 60°C for 5 min, then alkylated using 10 mM iodoacetamide, diluted to 0.1% RapiGest and finally trysinized overnight at 30°C. After SPE clean‐up, 2 μg of each sample was injected onto an Orbitrap Fusion mass spectrometer (Thermo‐Fisher Scientific) via a 50 cm long 75 μm column using a 6 h gradient delivered by a Dionex U‐3000 LC system (Thermo‐Fisher Scientific). Data were acquired with a resolution of 120 000 in MS1 and a scan range of 400–1600. Peptide ions were fragmented by HCD (35% collision energy) with a resolution of 15 000, an AGC target of 50 000, and a maximum injection time of 60 ms. The whole duty cycle was set to 2.5 s during which the instrument performed “top speed” analysis. Data were analyzed by label‐free quantitation using MaxQuant v1.5.0.12 21 and searched against a murine Uniprot‐Trembl database (51 372 entries; downloaded February 19 2014) and a list of common contaminants. This resulted in an identification of 3535 proteins (<1% FDR) and quantitation of 2510 proteins (present in two of the three replicates), providing the detailed analysis of the phagosome proteome so far (Supporting Information Table 1). A total of 290 and 226 proteins were significantly enriched (twofold, *p* < 0.05) on BMDM and RAW 264.7 phagosomes, respectively. Fifty eight BMDM and 17 RAW 264.7 proteins were considered unique to their cell line (a minimum of five quantified peptides in one and no quantitative values identified in the other cell type).

GO enrichment analysis of the proteins enriched on RAW 264.7 phagosomes compared to BMDM (Fig. 2A) includes ribosomes and translation proteins, which are probably overrepresented as RAW 264.7 is an actively proliferating cell line compared to BMDMs. Moreover, the vATPase complex is strongly enriched, although our functional data (Fig. 1B) showed a clear delay of the acidification in RAW 264.7. Interestingly, two vATPase subunits, ATP6v0a1 and ATP6ap2 (Renin receptor), are more abundant on BMDM phagosomes (Fig. 2B), suggesting they have an important role in the regulation of the acidifying complex. BMDM phagosomes have protein complexes enriched in the innate immune response, such as TLR 9 and TLR13 as well as complement receptors. Furthermore, they have strongly enriched integrin complexes (including integrins alpha‐5, alpha 6, beta‐1, beta‐2, and beta‐5) and express higher levels of lectins such as mannose receptor 1 (MRC1), galectins 1, 3, 8, and 9 as well as chitinase‐like protein 3 (Chil3/Ym1) and Sialoadhesin (Siglec1). Interestingly, BMDMs express also four times more Clec10a (Mgl1) which has been used with MRC1 and Ym1 as markers for alternatively activated macrophages 22. RAW 264.7, on the other hand, express higher levels of Clec4e (Mincle), Clec4a (Dcir), and Clec7a (Dectin 1), as well as a fourfold higher amount of interferon‐γ receptor, suggesting that the basal activation state of RAW 264.7 cells is considerably more proinflammatory compared to BMDMs.

**Figure 2:**
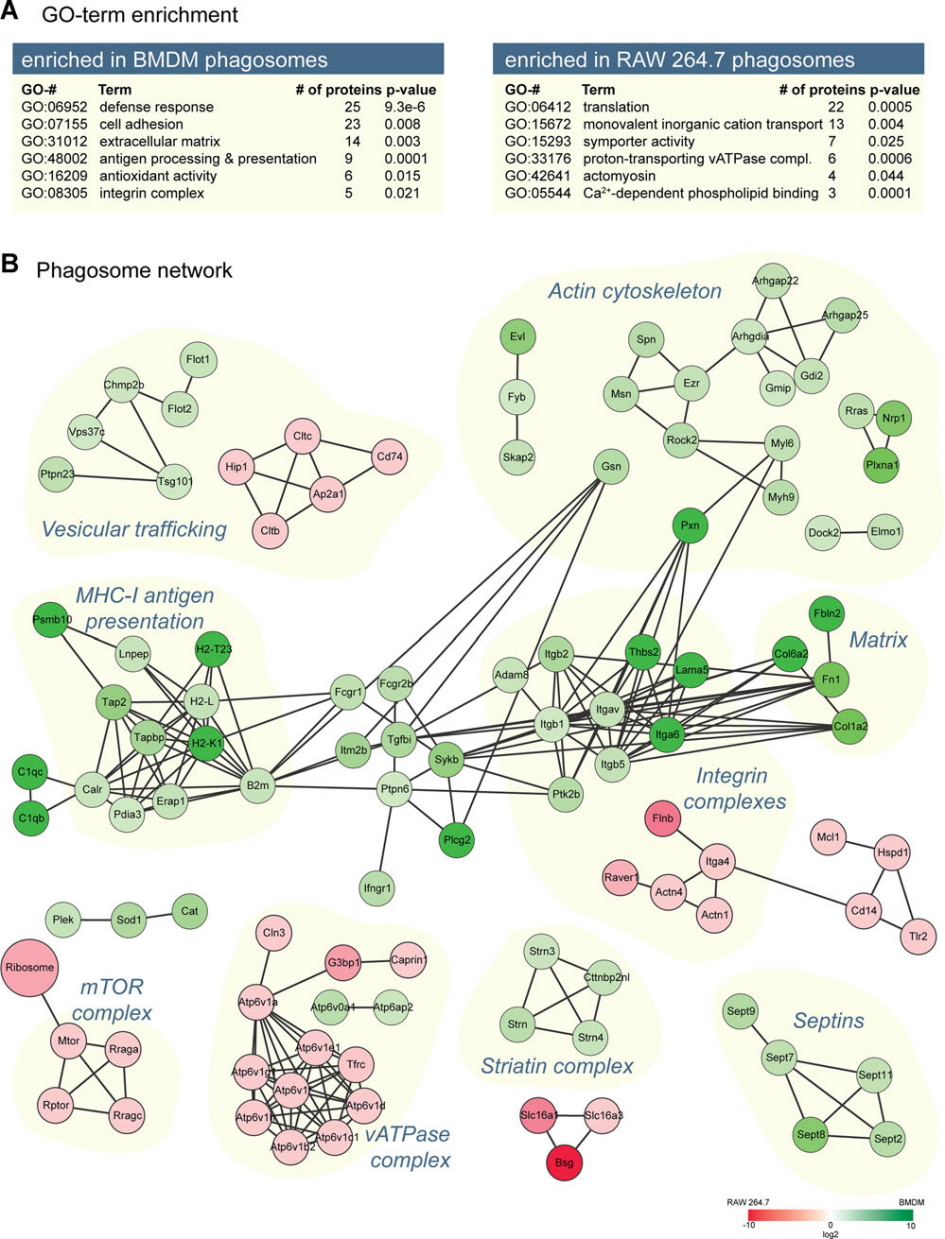
GO‐term enrichment and phagosome network analyses. (A) Selected GO terms of proteins enriched in the phagosome proteomes isolated from either BMDM or RAW 264.7 cells. (B) The phagosome network analysis shows cellular processes that were significantly enriched in the phagosomal proteome of BMDM (green) or decreased (red) compared to RAW 264.7 cells. The connection (gray edges) between nodes represents experimentally proven physical interaction between proteins.

Next, we performed a STRING analysis 23 of the proteins enriched in BMDMs and RAW 264.7 cells (Fig. 2B). This showed that various protein complexes are significantly enriched on BMDM phagosomes, including septin 2, 7, 8, 9, and 11, which have recently been shown to be important for phagosome formation 24, several complexes of cytoskeleton proteins associated with ezrin, moesin and Rock2 and Evl 25, proteins of the ESCRT complexes 26, the striatin complex 27 that has not previously been shown to locate to the phagosome, the above‐mentioned integrin complex, and the entire antigen crosspresentation complex associated with Tap2. One would therefore expect considerable differences in the ability to cross‐present antigens via MHC class I between RAW 264.7 cells and BMDMs.

We validated our proteomics results independently using Western blotting for a number of proteins (Fig. 3A). In RAW 264.7 cells, we confirmed a strong phagosomal enrichment of annexin‐V representing a larger number of annexins, indicative of a higher phospholipid content on RAW 264.7 phagosomes. Our data further show that Siglec1 is indeed only expressed in BMDMs. Moreover, we noticed that Syk, an important kinase for phagocytosis through various receptors 28, is located strongly to the phagosome in BMDMs, while it is not enriched in RAW 264.7 cells, with the majority being found in the total cell lysate. We further validated this by fluorescence microscopy (Fig. 3B), showing strong enrichment on BMDM phagosomes for Siglec‐1 and specific translocation for Syk.

**Figure 3:**
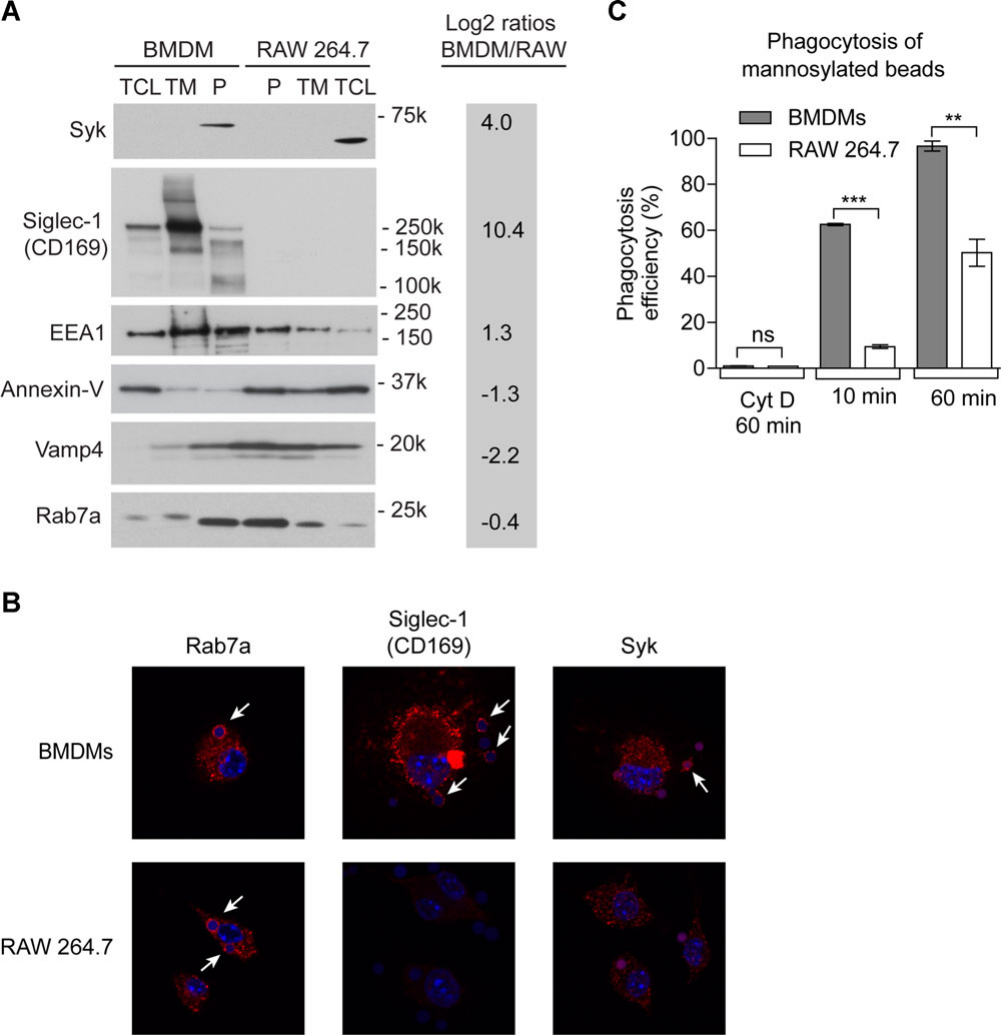
Validation of the proteomics results. (A) Western blotting analysis of six proteins selected from the proteomics data. P, phagosome; TM, total membrane extract; TCL, total cell lysate. Log2 ratios of proteomics results are given for convenience. (B) Fluorescence microscopy of Rab7a, Siglec‐1, and Syk proteins showed much stronger expression of Siglec‐1 in BMDMs than RAW 264.7 cells. Rab7a was localized to the phagosome in both cell types but Siglec‐1 and Syk were only associated with the phagosome in BMDMs. (C) The efficiency of phagocytosis of mannan‐coated beads is strongly increased in BMDMs compared to RAW 264.7 cells, due to the much higher expression of mannose receptor 1 (MRC1). Error bars represent standard deviation, pair‐wise *t*‐test comparison with ***p < 0.0001 and **p < 0.001.

Finally, as discussed above, we identified a >1000‐fold larger amount of MRC1 on phagosomes from BMDMs compared to RAW 264.7. This led us to investigate if uptake of beads coated with the polysaccharide mannan (Dill et al., submitted), which is taken up through mannose receptor 29, was affected between the two cell types. Figure 3C indeed shows an even stronger difference in phagocytic uptake of mannosylated beads than uncoated beads (Fig. 1A), indicating that MRC1 enhances the uptake of these beads in BMDMs.

In conclusion, we provide the largest dataset of the murine phagosome proteome published so far, which will be an important resource for researchers in the phagocytosis and macrophage field. Furthermore, our data indicate that several phagosomal functions such as acidification and proteolysis are significantly reduced in the RAW 264.7 cell line compared to BMDMs from C57/BL6 mice. By showing the molecular differences and similarities on the phagosome of these cell types, we allow researchers to choose the best model for their protein of interest.

*The authors have declared no conflict of interest*.

## Supporting information

As a service to our authors and readers, this journal provides supporting information supplied by the authors. Such materials are peer reviewed and may be re‐organized for online delivery, but are not copy‐edited or typeset. Technical support issues arising from supporting information (other than missing files) should be addressed to the authors.

Table S1

Supplement Information
