# Supplementary material for: High‐resolution quantitative proteome analysis reveals substantial differences between phagosomes of RAW 264.7 and bone marrow derived macrophages
Source: Proteomics. 2015 Feb 5;15(18):3169–74. doi: 10.1002/pmic.201400431 (PMC4833182; doi:10.1002/pmic.201400431)
Supplement: Supplementary file 2 — Supplement Information [file PMIC-15-3169-s002.pdf]

Supplementary Methods

Bone marrow-derived macrophages (BMDM) culture and phagosome isolation

Bone marrow cells were isolated from femurs of 8–12-wk-old C57Bl/6 female mice and differentiated into macrophages as described previously [1]. Briefly, bone marrow cells were cultured in tissue culture plastic for three days and then transferred into 10 cm Petri dishes and differentiated into macrophages for seven days in Dulbecco's minimal essential medium (DMEM, Gibco) supplemented with Foetal Bovine Serum (10% FBS, Labtech), L-glutamine (2 mM, Gibco), penicillin (100 U/ml, Gibco), streptomycin (100 µg/ml, Gibco), and 20% L929 cell conditioned medium as a source of M-CSF.

Phagosomes were isolated as described previously [2, 3]. Polystyrene particles of 0.8 µm (Estapor/Merck) were phagocytosed by macrophages for a 30 minute pulse, after which cells were subsequently washed and lysed. Phagosomes were isolated using sucrose gradients. Cells culture and phagosome isolation were performed in triplicate.

Beads conjugation and phagosomal functional assays

Phagocytosis assays were performed using fluorophore coated beads. Carboxylate silica beads (3 µm, Kisker Biotech) were coated with avidin after activation by EDC/NHS [250 mM 1-Ethyl-3-(3-dimethylaminopropyl)carbodiimide and 350 mM N-hydroxysuccinimide, Sigma]. Succinimide-Alexa Fluor 488 (Molecular Probes) was conjugated to avidinylated beads. For mannosylated beads, Mannan (Sigma) was oxidized in 2 mM sodium meta-periodate (Pierce) in 100 mM sodium acetate pH 5.5 on ice for 30 minutes and desalted with Zeba columns (Pierce) as described by

manufacture. Oxidized mannan was incubated with 5 mM biotin-LC-hydrazide (Pierce) in PBS for 2 hours and desalted again. Fluorescent mannosylated beads were generated by incubating biotinylated mannan and Alexa Fluor 488 together with avidinylated beads.

The fluorogenic assays for phagosomal functions are adapted from the methods from previous work [4, 5]. For proteolysis and oxidative burst, DQ Green or Oxyburst Green BSA (Molecular Probes) were coupled, respectively, with avidin to beads as described above. Subsequently, Alexafluor 594 or 647 were conjugated to DQ Green or Oxyburst Green-avidinylated beads as control fluorophores. For acidification, BCECF (Molecular Probes) was conjugated to avidinylated beads.

BMDM were plated onto 96-well plates at a density of 1 x 10^5 cells/well. DQ Green BSA-coupled, BCECF-coupled or OxyBurst Green BSA-coupled beads were diluted 1:200 in binding buffer (1 mM CaCl2, 2.7 mM KCl, 0.5 mM MgCl2, 5 mM dextrose, 10 mM hydroxyethyl piperazine ethane sulfonate [HEPES] and 5% FBS in PBS pH 7.2) and incubated with BMDM for 3 min at room temperature. Beads were replaced with warm binding buffer, and real-time fluorescence was measured at 37°C using a SpectraMax Gemini EM Fluorescence Microplate Reader (Molecular Devices), set as maximal readings per well to allow reading time intervals of 2 min. Plots were generated from the ratios of signal/control fluorescence. Error bars indicate standard error from six replicates.

LC-MS/MS analysis and protein quantitation

Phagosome proteins were extracted using 1% sodium 3-[(2-methyl-2-undecyl-1,3-dioxolan-4-yl)methoxy]-1-propanesulfonate (commercially available as RapiGest,

Waters) in 50 mM pH 8.0 Tris, reduced with tris(2-carboxyethyl)phosphine (TCEP), alkylated by iodoacetamide (Sigma), and finally digested by Trypsin Gold (Promega).

Samples were analysed on an Orbitrap Fusion Tribrid mass spectrometer (Thermo-Fisher Scientific) coupled to an Ultimate 3000 UHPLC system with a 50 cm EasySpray Acclaim PepMap 100 analytical column (75 μm ID, 3 μm C18) in conjunction with a Pepmap trapping column (100 μm x 2 cm, 5 μm C18) (Thermo-Fisher Scientific). Six hour linear gradients were performed from 3% solvent B to 35% solvent B (solvent A: 3% acetonitrile, 0.1% formic acid; solvent B: 80% acetonitrile, 0.08% formic acid). For each run, sample of 2 μg was injected. Settings for data acquisition were: MS1 with 120,000 resolution, scan range of 400-1600, charge states 2-5, AGC target of 200,000, and dynamic exclusion of 60s with repeat count 1. Peptide ions were fragmented by HCD (35% collision energy) with a resolution of 15,000, an AGC target of 50,000, and a maximum injection time of 60 ms. The whole duty cycle was set to 2.5 s during which the instrument performed "top speed" analysis.

Data was analysed by label-free quantitation using MaxQuant v1.5.0.12 [6] and searched against a murine Uniprot-Trembl database (51,372 entries; downloaded February 19 2014) and a list of common contaminants. Label-free quantification was performed using MaxQuant Version v1.5.0.12 [6] with the following parameters: stable modification carbamidomethyl (C); variable modifications oxidation (M), acetylation (protein N-terminus), Glu->pyro-Glu; label-free quantitiation with a minimum ratio count 2; maximum 5 modifications per peptide, and 2 missed cleavages. Identifications were filtered at a 1% false-discovery rate (FDR). Quantification used only razor and unique peptides with a minimum ratio count of 2.

"Re-quantify" was enabled. "Match between runs" was used with alignment time window 20 min and match time window 0.7 min. LFQ intensities were used for data analyses.

Data analyses

Statistical analyses were performed in Perseus (v1.4.0.17) [7]. Protein ratios were generated from LFQ intensities, and significant changes determined by applying a one sample t-test ($p<0.05$) to the logarithmized protein ratios. The GO-term enrichment analysis was performed using Database for Annotation, Visualization and Integrated Discovery (DAVID, version 6.7). The significant enriched proteins (fold change >2, $p<0.05$) and unique proteins (a minimum of 5 quantified peptides in one and no quantitative values identified in the other cell type) were analysed against the background of all identified phagosomal proteins in the experiment.
Network analysis was performed by submitting a combined enriched and unique protein list to the STRING database [8] using only "experiments" and "database" data with a confidence higher than 0.400. Data was extracted and interesting complexes isolated using Cytoscape 3.0.2 [9].

Antibodies for immunoblotting

The primary antibodies were used: sheep anti-Siglec-1 (CD169) (RD Systems; AF5610), rabbit anti-Syk (Cell Signaling; 2712), rabbit anti-EEA1 (Cell Signaling, 2411), anti-annexin-V (Cell Signaling, 8555), rabbit anti-Vamp4 (Abcam; ab3348) and rabbit anti-Rab7a (Cell Signaling, 2094).

Immunofluorescence

BMDM were seeded on glass coverslips in 6-well plates at a density of 1×10^5 cells/well. Silica beads (3 μm, Kisker Biotech) were phagocytosed for 30 min by

using a dilution of 1:1000 in cell culture media. Following phagocytosis, cells were washed with PBS, fixed in 4% paraformaldehyde in PBS (Affymetrix), permeabilized by 0.02% NP-40 in PBS for 5 min and incubated with rabbit anti-Rab7a (1:300), sheep anti-Siglec-1 (CD169) (1:100) or rabbit anti-Syk (1:100) for 1 h. After washing, cells were incubated with AlexaFluor 594 conjugated secondary antibodies (1:500, Molecular Probes) for 30 min, mounted with ProLong® Gold anti-fade mountant containing DAPI and imaged using a Zeiss LSM 700 confocal microscope with a Plan Apochromat 100x/NA 1.46 objective. Laser intensity, pin-hole size and scan speed were kept the same for comparative samples (cell types).

References

[1] Weischenfeldt, J., Porse, B., Bone Marrow-Derived Macrophages (BMM): Isolation and Applications. *CSH Protoc* 2008, *2008*, pdb prot5080.

[2] Trost, M., English, L., Lemieux, S., Courcelles, M., *et al.*, The phagosomal proteome in interferon-gamma-activated macrophages. *Immunity* 2009, *30*, 143-154.

[3] Desjardins, M., Huber, L. A., Parton, R. G., Griffiths, G., Biogenesis of phagolysosomes proceeds through a sequential series of interactions with the endocytic apparatus. *J Cell Biol* 1994, *124*, 677-688.

[4] Yates, R. M., Hermetter, A., Taylor, G. A., Russell, D. G., Macrophage activation downregulates the degradative capacity of the phagosome. *Traffic* 2007, *8*, 241-250.

[5] Podinovskaia, M., VanderVen, B. C., Yates, R. M., Glennie, S., *et al.*, Dynamic quantitative assays of phagosomal function. *Curr Protoc Immunol* 2013, *102*, Unit 14 34.

[6] Cox, J., Mann, M., MaxQuant enables high peptide identification rates, individualized p.p.b.-range mass accuracies and proteome-wide protein quantification. *Nat. Biotechnol.* 2008, *26*, 1367-1372.

[7] Cox, J., Mann, M., 1D and 2D annotation enrichment: a statistical method integrating quantitative proteomics with complementary high-throughput data. *BMC Bioinformatics* 2012, *13 Suppl 16*, S12.

[8] Franceschini, A., Szklarczyk, D., Frankild, S., Kuhn, M., *et al.*, STRING v9.1: protein-protein interaction networks, with increased coverage and integration. *Nucleic Acids Res.* 2013, *41*, D808-815.

[9] Shannon, P., Markiel, A., Ozier, O., Baliga, N. S., *et al.*, Cytoscape: a software environment for integrated models of biomolecular interaction networks. *Genome Res.* 2003, *13*, 2498-2504.
